# A near infrared light-triggerable modular formulation for the delivery of small biomolecules

**DOI:** 10.1186/s12951-019-0530-y

**Published:** 2019-09-16

**Authors:** Vitor Francisco, Miguel Lino, Lino Ferreira

**Affiliations:** 1 CNC-Center for Neurosciences and Cell Biology, University of Coimbra, 3004-517 Coimbra, Portugal; 2 Faculty of Medicine, University of Coimbra, 3000-548 Coimbra, Portugal

**Keywords:** Modular platform, NIR light trigger, Gold nanorods, Host–guest chemistry, Temperature control

## Abstract

**Background:**

Externally triggered drug delivery systems hold considerable promise for improving the treatment of many diseases, in particular, diseases where the spatial–temporal release of the drug is critical to maximize their biological effect whilst minimizing undesirable, off-target, side effects.

**Results:**

Herein, we developed a light-triggerable formulation that takes advantage of host–guest chemistry to complex drugs functionalized with a guest molecule and release it after exposure to near infrared (NIR) light due to the disruption of the non-covalent host–guest interactions. The system is composed by a gold nanorod (AuNR), which generates plasmonic heat after exposure to NIR, a thin layer of hyaluronic acid immobilized to the AuNR upon functionalization with a macrocycle, cucurbit[6]uril (CB[6]), and a drug functionalized with a guest molecule that interacts with the macrocycle. For proof of concept, we have used this formulation for the intracellular release of a derivative of retinoic acid (RA), a molecule known to play a key role in tissue development and homeostasis as well as during cancer treatment. We showed that the formulation was able to conjugate approximately 65 μg of RA derivative per mg of CB[6] @AuNR and released it within a few minutes after exposure to a NIR laser. Importantly, the bioactivity of RA released from the formulation was demonstrated in a reporter cell line expressing luciferase under the control of the RA receptor.

**Conclusions:**

This NIR light-triggered supramolecular-based modular platform holds great promise for theranostic applications.

## Introduction

Intracellular delivery of biomolecules is quintessential for the modulation of cellular processes, cellular reprogramming and gene editing [1, 2]. Many of these molecules act at the intracellular level and thus require transporters to cross the cell membrane. Several nanocarriers have been developed to facilitate the intracellular transport of biomolecules [3–5]. For example, liposomes and polymer technologies have been frequently used as drug delivery systems [6, 7]. More recently, light-triggerable nanocarriers have been proposed as an alternative to other type of synthetic carriers given their potential to release the biomolecule of interest in a spatial–temporal controlled manner thus maximizing the therapeutic effect whilst reducing the negative side-effects [8–10]. Indeed, we have recently developed several light-triggerable nanocarriers that had the capacity to orchestrate the intracellular delivery of proteins [11], miRNAs [9] and small molecules [12]. Despite these advances, the proposed strategies relied on the complex and time-consuming synthesis of photo-cleavable linkers for the attachment of biomolecules with different chemistries.

By taking advantage of noncovalent interaction between host and guest molecules, supramolecular chemistry has been shown to be a promising approach for the controlled release of drugs [13]. Cucurbit[*n*]urils (CB[*n*]) are a family of macrocycles, with a pumpkin-shaped structure composed of *n* glycoluril units (*n *= 5–8, 10, 14) bridged by methylene groups, which form stable host–guest inclusion complexes with a wide variety of guest molecules [14, 15]. The remarkable selectivity and affinity of this class of macrocycles is driven by a combination of ion dipole interactions, hydrogen bonds, and hydrophobic effects [16]. Unfortunately, the use of supramolecular chemistry, in particular the one based on CB[n], is largely unexplored for the design of light-triggerable drug-releasing systems [17, 18].

**Fig. 1:**
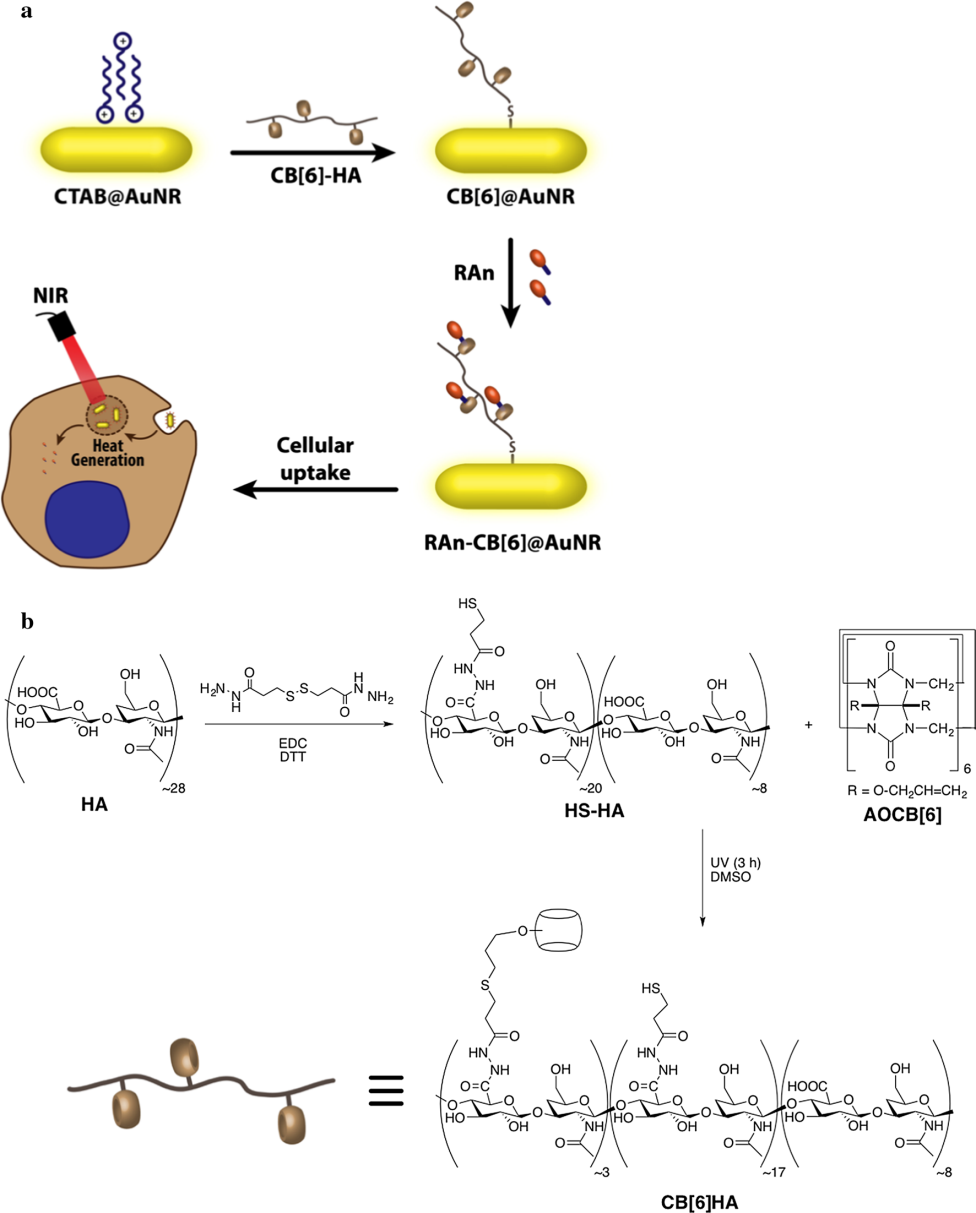
**a** Schematic representation of the preparation of RAn-CB[6] @AuNR formulation for the intracellular delivery of RA after NIR activation. **b** Scheme for the synthesis of CB[6] HA conjugate. HA (8–15 kDa) was reacted with 3,3´-dithiobis(propanoic hydrazine) (DTP) in the presence of 1-ethyl-3-[3-(dimethylamino)propyl]carbodiimide (EDC) and then dithiothreitol (DTT) to obtain 70% thiolated HA (HS-HA). Next, HS-HA was reacted with (allyloxy)_12_CB[6] in DMSO for 3 h under UV light to obtain CB[6] HA (DS = 8 ± 1 mol %)

Here, we report a light-triggerable system that is based on a light-triggerable antenna, formed by a gold nanorod (AuNR), coated with a polymeric coating conjugated with cucurbit [6] uril (CB[6]) (host molecule) (Fig. 1a). This system can be used to immobilize any biomolecule conjugated with guest molecules displaying variable affinity to CB[6]. In contrast to previously reported light-triggerable systems, the current approach relies on a core system that regardless of the chemistry of the biomolecule to be delivered, does not change. The interaction of the biomolecules to the nanocarrier is solely dictated by the chemistry of the guest molecule attached to the biomolecule, making it a simple system for the delivery of multiple biomolecules without requiring extensive chemical modifications. The selection of AuNRs was based on the fact that these nanomaterials (i) have been widely used for drug delivery [19], bioimaging [20] and hyperthermia [21], due to their simple synthesis, facile surface modification, adaptable conjugation with biomolecules and tunable optical properties [19, 22] and (ii) they can absorb NIR light, which has higher tissue penetration than UV/blue light radiation, and convert it in photothermal energy [23]. Based on these premises, we hypothesized that the increase in local temperature could reduce the interaction between the host and the guest releasing the later in a controlled manner. For proof of concept, we have used all-trans retinoic acid (RA) modified with guest molecules as a model biomolecule. RA controls the biological activity of neural stem cells [24, 25], leukemia cells [26], and endothelial progenitor cells [27]. In addition, RA is a powerful anti-cancer agent, currently being used in several types of cancers [28–31], such as acute promyelocytic leukemia (APL) [32]. Our results showed that the light-triggerable formulation provided an excellent platform to control the release of RA derivatives and ultimately elicit a local response at the cellular level.

## Experimental section

### NMR spectrometry

^1^H NMR spectra were recorded on a Bruker Avance III spectrometer at 400 MHz. The spectra were recorded in D_2_O using a pulse of 90° and relaxation delay of 4.0 s. The residual solvent (non-deuterated) was used as the internal reference.

### FTIR analyses

FTIR spectra were recorded with a Nicolet Magna-IR 550 spectrometer (Madison, WI). The dry samples were powdered, mixed with KBr, and pressed into pellets under reduced pressure. The FTIR spectra were obtained by recording 128 scans between 4000 and 650 cm^−1^ with a resolution of 2 cm^−1^.

### UV–Vis and fluorescence spectroscopy

UV–Vis and fluorescence analysis was recorded on a Synergy H1 microplate reader.

### Microcalorimetry

The microcalorimetric titrations were performed using an isothermal titration microcalorimeter (VP-ITC, Malvern) at atmospheric pressure and at a specific temperature. In each run, a solution of guest in a 0.27 mL syringe was sequentially injected with stirring at 351 rpm in a solution of host in the sample cell. Each solution was degassed and thermostated by using a ThermoVac accessory before titration. In all experiments the first injection was rejected to eliminate diffusion effects of material from the syringe onto the calorimetric cell. The ITC experiments were analyzed with the software ORIGIN (provided by Malvern Inc.) by using the “one set of binding sites” model to simultaneously compute the binding stoichiometry (*N*), complex stability constant (*K*_a_), standard molar reaction enthalpy (Δ*H*°) and standard deviation from the titration curve.

### Confocal microscopy

Images were acquired using a Zeiss LSM 710 confocal microscope (Carl Zeiss, Jena, Germany) with a 40× objective/1.4 numerical aperture oil PlanApochromat immersion lens. Alexa-fluor 488 fluorescence was detected using the 488 nm laser line of an Ar laser and an LP 505 filter. TRITC fluorescence was detected using a 561 nm HeNe laser (1 mW) and an LP 560 filter and Alexa-fluor 633 fluorescence was detected using a 633 nm HeNe. Z-stacks were acquired to confirm the intracellular localization of TRITC labelled AuNRs. The thickness of the slices and the interval between slices were set to 0.7 µm. After irradiation, cells were deposited onto a glass slide using cytospin centrifugation (800 rpm, 5 min), fixed with 4% PFA for 20 min and stained for confocal microscopy. Cell membrane was stained with monoclonal mouse anti-human CD45 antibody, diluted 1:50 (R&D Systems, MAB1430) and using Alexa Fluor-488 goat anti-mouse igG as secondary antibody, diluted 1:1000 (Life technologies, A11001). For staining early endosomes, cells were permeabilized with 0.1% TRITON X-100 for 10 min and stained with rabbit anti-EEA1 monoclonal antibody (Cell Signaling Technology, 3288), diluted 1:100. Alexa Fluor-633 goat anti-rabbit (Life Technologies, A21070), diluted 1:1000 was used as secondary antibody. Cell nuclei were stained with DAPI.

### Synthesis of RA conjugates

The conjugates were prepared using a procedure described elsewhere [33–35]. The synthesis of conjugate RA1 ((2E,4E,6E,8E)-N-(4-(1H-imidazol-1-yl)phenyl)-3,7-dimethyl-9-(2,6,6-trimethylcyclohex-1-en-1-yl)nona-2,4,6,8-tetraenamide) was performed as follows: a solution of 4-(1H-Imidazol-1-yl)aniline (IA) (51.5 mg, 0.32 mmol, Sigma-Aldrich) and *N,N*-diisopropylethylamine (DIPEA) (50 µL, 0.29 mmol, Sigma-Aldrich) in 1.0 mL of dimethylformamide (Alfa Aesar) was added dropwise to an ice-cold suspension of RA (121.5 mg, 0.40 mmol, Sigma-Aldrich), 1-hydroxybenzotriazole hydrate (HOBt) (61.2 mg, 0.45 mmol, Sigma-Aldrich), *N,N,N′,N′*-tetramethyl-O-(1H-benzotriazol-1-yl)uronium hexafluorophosphate (HBTU) (171.8 mg, 0.45 mmol, Sigma-Aldrich) and DIPEA (230 µL, 1.33 mmol) in 2.5 mL of dimethylformamide and the resulting solution was stirred overnight at room temperature. Volatile components were removed under vacuum before column chromatography purification and, upon evaporation of the solvents, pure compound RA1 (93 mg, 65%) was obtained, as a yellow solid.

The synthesis of conjugate RA2 ((2E,4E,6E,8E)-N-(6-aminohexyl)-3,7-dimethyl-9-(2,6,6-trimethylcyclohex-1-en-1-yl)nona-2,4,6,8-tetraenamide) and RA3 ((2E,4E,6E,8E)-N-(3-((4-((3-aminopropyl)amino)butyl)amino)propyl)-3,7-dimethyl-9-(2,6,6-trimethylcyclohex-1-en-1-yl)nona-2,4,6,8-tetraenamide) was performed as follows: to an ice-cold solution of RA (100 mg, 0.33 mmol) in dry tetrahydrofuran (THF) (1 mL, Sigma-Aldrich) was added sequentially N-hydroxysuccinimide (NHS) (57.5 mg, 0.5 mmol, Sigma-Aldrich) and *N,N′*-dicyclohexylcarbodiimide (DCC) (82.5 mg, 0.4 mmol, Sigma-Aldrich) and the resulting mixture was stirred overnight at room temperature. The precipitated dicyclohexylurea was filtered off (WLP filter funnel, porosity 4) and washed several times with EtOAc (Fisher Chemical). The combined filtrates were washed sequentially with ice-cold 5% aqueous (aq.) solution of sodium bicarbonate (Sigma-Aldrich), water and once with brine. The organic layer was dried over Na_2_SO_4_ (Panreac), followed by filtration and evaporation of the solvent in a rotary evaporator (T_water bath_ = 45 °C, Vacuubrand PC 500 series) left a yellow solid, succinimidyl all-*trans*-retinoate. Next, to an ice-cold solution of 1,6-hexanediamine (HMA) (0.05 g, 0.43 mmol, Sigma-Aldrich) or spermine (0.1 g, 0.5 mmol, Sigma-Aldrich) in dichloromethane (DCM) (5 mL, Fisher Chemical) a solution of succinimidyl all-*trans*-retinoate (0.05 g, 0.125 mmol) in DCM (5 mL) was added dropwise during 30 min and the resulting mixture was stirred overnight at room temperature. The reaction mixture was diluted with dichloromethane and washed sequentially with an ice-cold 5% aqueous solution of sodium bicarbonate and brine. Next, the mixture was dried overnight with Na_2_SO_4_, followed by filtration, evaporation of the solvent and purified by HPLC to obtain pure products RA2 (0.22 g, 46%) and RA3 (0.24 g, 40%).

### Compounds purification

Compound RA1 was purified by column chromatography packed with silica gel 60A (60–200 µm, Acros) and the purification process monitored by thin layer chromatography (TLC) on aluminium plates coated with 60F_254_ (0.2 mm, Merck). Spots were visualized with UV light at 254 nm or ninhydrin (Sigma-Aldrich). The eluent system used was hexane/ethyl acetate (6:4, v/v). Analytical RP-HPLC was performed on a Shimazo Prominence-I LC-2030 C 3D and elution of the compounds was monitored by absorbance at 254 nm. Compound purity was assessed using a XBridge C18 3.5 µm 4.6 × 250 mm column (Waters) and a linear gradient of 10–60% acetonitrile (containing 0.08% TFA) in water (containing 0.08% TFA) for compounds RA2 and RA3 over 40 min at a flow rate of 1 mL/min.

### Synthesis and characterization of thiolated hyaluronic acid (HA)

The synthesis of thiolated HA (HS-HA) was prepared as described elsewhere [36]. Briefly, HA sodium salt (0.2 g, 0.5 mmol, Mw = 8–15 kDa, Carbosynth Limited, Berkshire, UK) was dissolved in 20 mL of water followed by the addition of 3,3′-dithiobis(propanoic hydrazide) (DTP) (0,238 g, 1.0 mmol, Frontier Scientific) while stirring. The pH of the mixture was adjusted to 4.75 with HCl (Merck). Next, 1-ethyl-3-[3-(dimethylamino)propyl]carbodiimide (EDC) (0.192 g, 1.0 mmol, Sigma-Aldrich) was added in solid form and the mixture stirred for 2.5 h. The reaction was stopped by increasing the pH to 7.0 with NaOH (Merck). Then, dithiothreitol (DTT) (1.0 g, 6.5 mmol, Acros) was added, the pH increased to 8.5, and the mixture was stirred (250 rpm) overnight. After decreasing the pH of the mixture to 3.5 with HCl, the reaction product was purified by dialysis (Mw cutoff = 2 kDa) against diluted HCl, pH = 3.5, and lyophilized for 48 h to yield HS-HA. The degree of substitution in HS-HA was determined by ^1^H NMR (ratio between methylenes of DTP and *N*-acetyl methyl protons of HA) and by free thiol content as measured by an Ellman’s test.

### Synthesis of CB[6] HA conjugate

HS-HA with a thiol content of 70% (5 mg, 10.9 µmol) was added to a solution of tris(2-carboxyethyl)phosphine hydrochloride (TCEP) (9 mg, 31.8 µmol, Sigma-Aldrich) in anhydrous DMSO (2 mL) in a quartz cuvette. Perallyloxycucurbit [6] uril potassium sulfate (AOCB[6]) (10 mg, 5.5 µmol, Strem Chemicals) was added to the mixture and then irradiated with UV light (365 nm, 100 W) for 6 h. The unreacted AOCB[6] and TCEP were removed by dialysis for 48 h (MWCO 12–14 kDa) against DMSO and water successively. The degree of CB[6] modification in the HA was assessed by ^1^H NMR through the ratio between the peaks corresponding to allyloxy groups of AOCB[6] (5.25–6.15 ppm) and *N*-acetyl methyl protons of HA (2.0 ppm).

### Preparation of AuNRs

AuNRs were prepared following the seed-mediated method [37]. The seed solution was prepared by the addition of ice-cold sodium borohydride (NaBH_4_) (10 mM, 0.3 mL, Sigma-Aldrich) to a solution of hexadecyltrimethylammonium bromide (CTAB) (0.1 M, 5 mL, Sigma-Aldrich) containing chloroauric acid (HAuCl_4_.aq) (0.25 mM, Sigma-Aldrich). The solution was stirred for 2 min and then kept at 25 °C for 8 min. The growth solution was prepared by the sequential addition of silver nitrate (AgNO_3_) (5 mM, 3.2 mL), HAuCl_4_· × H_2_O (50 mM, 2 mL) and ascorbic acid (0.1 M, 1.5 mL, Sigma-Aldrich) to a CTAB solution (0.1 M, 200 mL, Sigma-Aldrich), mixing gently after each step. Finally, the seed solution was kept at 28 °C for 2 h and then centrifuged twice at 9000*g* (20 min) to purify the AuNRs.

### Characterization of the AuNRs

AuNRs size were measured with a PHILIPS CM-12 transmission electron microscope at 100 kV. A drop of dispersed AuNRs (lyophilized powder) into water was spread on a 200-mesh copper grid coated with a Formvar film, and the extra droplet was instantly wiped off by filter paper. The dried grid was then examined under an electron microscope. Additionally, AuNRs were analysed by photon correlation spectroscopy (PCS) using quasi-elastic light scattering equipment (Zeta-Pals™ Zeta Potential Analyzer, Brookhaven Instruments Corp., Holtsville, NY) and ZetaPlus™ Particle Sizing Software (version 4.03). A AuNR suspension before and after functionalization (2 mL, 50 µg/mL in water of molecular biology) was added to a cuvette and allowed to stabilize for 10 min and then analysed (3 times) at room temperature. In some experiments, the cuvette was then exposed to NIR light (780 nm, 2 W/cm^2^) for 3 min and the values of NP diameter and NP counts (Kcps) were recorded. The zeta potential of NPs was determined in a 1 mM KCl, at 25 °C (2 mL, 50 µg/mL). All data were recorded with at least 5 runs (in triplicate) with a relative residual value (measure of data fit quality) of 0.03. Nanoparticles tracking analysis (NTA) measurements were performed using the NS300 Particle Measuring Instrument from NanoSight Ltd (NanoSight, GB) containing a sample chamber of 0.25 mL, a 532 nm laser and a camera sCMOS. For each run, about 1 mL of the sample was manually injected into the chamber at 37 °C. Five videos of 30 s duration were taken, with a frame rate of 30 frames per second. For data capturing and data evaluation, the NTA 3.2 analytical software was used (NanoSight Ltd). In the analysis, the mode size (main peak), mean size and its standard deviation values were obtained.

### Temperature profile of AuNR suspensions

The temperature variation upon irradiation a suspension of RAn-CB[6] @AuNR (10 or 50 µg/mL in RPMI medium) was recorded using an infrared camera (FLIR SC5650). The AuNR suspension in a 24 well plate was irradiated with a continuous wave NIR laser (780 nm) at a power density of 2 W/cm^2^. The volume used for each well was fixed at 0.4 mL and RPMI medium was used as a control. The laser beam was collimated and expanded to a circular Gaussian beam with a diameter of about 9 mm.

### Preparation of CB[6] @AuNR complex

Functionalization of CTAB@AuNR was performed through ligand exchange with CB[6] HA in water. CB[6] HA solution (500 µL, 2 mg/mL) was added to the AuNR suspension (2 mL, 0.3 nM), stirred for 4 h, and CB[6] @AuNR was purified by centrifugation at 10,000*g* for 25 min twice and finally dispersed in cell culture RPMI-1640 medium ((Gibco) supplemented with 10% fetal bovine serum (Gibco) and 100 U/mL PenStrep (Lonza). The average number of CB[6] HA molecules bound to a single AuNR was calculated indirectly, i.e. by the quantification of CB[6] HA not bounded to AuNRs (in the supernatant after centrifugation) using a colorimetric assay with anthrone/sulfuric acid [38].

### Drug loading in AuNRs

The RA conjugates concentration in the supernatant was determined by a UV–vis spectrophotometer at 350 nm to calculate the drug loading content. The drug loading content and entrapment efficiency were calculated by the following equations: Loading content = (weight of drug in CB[6] @AuNR)/(weight of CB[6] @AuNR); Entrapment efficiency = (weight of drug in CB[6] @AuNR)/(initial weight of drug). In the in vitro drug release experiment, a suspension of RAn-CB6@AuNR (1 mL, 10 μg/mL) was agitated at 37 °C followed by centrifugation at 10,000*g* during 25 min at 37 °C. The supernatant was collected and the amount of released drug was determined by measuring the absorption at 350 nm using a UV–vis spectrometer and calculated by the linear regression equation y = 0.1018 × (*R*^2^ = 0.99) for RA1, y = 0.102 × (*R*^2^ = 0.99) for RA2 and y = 0.0995 × (*R*^2^ = 0.99) for RA3.

### Cell Culture

Human bone marrow acute promyelocytic leukemia NB4 cells, kindly provided by Dr. Arthur Zelent (Institute of Cancer Research, Royal Cancer Hospital), were cultured in supplemented RPMI-1640 medium (Gibco) [10% fetal bovine serum (Gibco) and 100 U/mL PenStrep (Lonza)] in a CO_2_ incubator at 37 °C, 5% CO_2_ in a humidified atmosphere. NB4-RARE cell line generation was obtained as previously reported by us [26]. Cells were passaged every 2–3 days and used for experiments between passage 2 and 8.

### Release studies of RA from RAn-CB[6] @AuNRs

The release of RA from RAn-CB[6] @AuNRs was evaluated by a biological assay, using a reporter cell line (NB4-RARE cells) that expresses luciferase after the activation of the RA receptor [26]. Prior to cell transfection, a RAn-CB[6] @AuNR suspension (50 µg/mL) in RPMI medium was irradiated outside the cells with NIR light for 2 min (780 nm, 2 W/cm^2^), centrifuged (25 min, 10,000*g*, 37 °C), collected the supernatant (1 mL) and resuspended the pellet in fresh medium. NB4-RARE cells (2.0 × 10^4^ cells/well) were plated in V-shaped 96-well plates and immediately cultured with RA1 (1 μM), RA2 (1 μM), supernatant of RA1 and RA2 and resuspended pellet (50 µg/mL) for 4 h in supplemented RPMI-1640 medium. The cells were then washed with PBS and centrifuged (250*g*, 5 min) to remove non-internalized AuNRs. Following transfection, cells were cultured for 20 h in supplemented RPMI-1640 medium. After this time, cells were centrifuged and then resuspended in cell lysis buffer (60 μL; the buffer was composed by 2.5 mM of magnesium chloride, 33 mM dl-dithiothreitol, 0.1 mM ethylenediaminetetraacetic acid, 20 mM tricine, 2.5 mM magnesium sulfate and 1% Triton X-100). To allow complete lysis of the cells, the plate was kept on ice under agitation for 15 min and subsequently stored at − 80 °C for the amount of time necessary to freeze the samples. Finally, the plate was thawed at slow rate on ice and the luciferase luminescence was quantified, in a well-by-well mode, in a microplate luminometer reader LumiStar Galaxy (BMG Labtech), at 37 °C with constant agitation, by adding, with an injector, the reading buffer (100 μL; the reading buffer was formed by 0.5 mM luciferin, 0.5 mM ATP, 2.5 mM of magnesium chloride, 33 mM DL-dithiothreitol, 0.1 mM ethylenediaminetetraacetic acid, 20 mM tricine and 2.5 mM magnesium sulfate) to the sample (50 μL). Protein was quantified using Pierce BCA Protein Assay Kit (Thermo Scientific) in order to normalize the luminescence signal to the mass of protein in each condition. All measures were performed in triplicate.

### CB[6] @AuNR internalization studies

The amount of RAn-CB[6] @AuNR internalized by NB4 cells was monitored by inductively coupled plasma mass spectroscopy (ICP-MS). The intracellular levels of Au were measured before and after cell exposure. NB4 cells (20 × 10^3^ cell/well) were plated in 96 well plates and incubated with RPMI-1640 medium from 1 to 24 h with RAn-CB[6] @AuNRs (50 µg/mL). After incubations, the cells were centrifuged and washed with PBS (three times), followed by the addition of an aqueous solution of nitric acid [1 mL, 69% (v/v)]. The intracellular concentration of Au was quantified by ICP-MS and normalized per cell. The estimation of AuNRs was done based on controlled standard solutions.

### Cytotoxicity of CB[6] @AuNR

To assess the cytotoxicity of AuNRs, NB4 cells were incubated for 4 h in a 96 well plate (20 × 10^3^ cell/well) with variable amount of RAn-CB[6] @AuNRs (concentration between 10 and 100 µg/mL). After incubation, cells were washed twice with PBS by centrifugation (300*g*–5 min) to remove non-internalized AuNRs. Then, cells were left in the incubator with RPMI medium for additional 20 h and the ATP production was measured by a Celltiter-Glo Luminescent Cell Viability Assay (Promega, USA) performed according to the manufacturer´s instructions.

### Labelling of CB[6] @AuNR with TRITC

Thiol-PEG-amine 5 kDa (100 nmol, Creative PEGworks) was reacted with tetramethylrhodamine isothiocyanate (TRITC) (200 nmol, Sigma-Aldrich) in 1 mL of 100 mM carbonate buffer at pH 9.2 for 2 h at room temperature. Then CB[6] @AuNR (500 µL, 0.2 nM) were incubated overnight with thiol-PEG-TRITC in a molar ratio of 1:1000. The excess fluorophore was removed in two steps of centrifugation at 10,000*g* for 25 min.

### NB4 differentiation assay

Myelocytic differentiation of NB4 cells was assessed by quantifying CD11b^+^ population using flow cytometry. NB4 cells (5000 cells/condition) were plated in a 96-well plate and cultured for 4 h with RAn-CB[6] @AuNR. The cells were then washed by centrifugation (300*g*–5 min) to remove non-internalized AuNRs and irradiated or not with NIR light. After 3 days, NB4 cells were stained with CD11b (clone ICRF44—PE mouse anti-human antibody from BD Pharmingen) for 10 min at room temperature in the dark. Following incubation, the cells were washed twice with 1 mL of PBS 1X and centrifuged for 5 min at 300*g*. Then, the cells were suspended in 100 µL of PBS 1X and characterized by a BD Accuri C6 flow cytometer (5000 events were recorded). Unstained cells were used to set the cutoff for the expression of the different markers.

### Statistical analysis

All the experiments were performed at least 3 times. Where applicable, the significance of variability between the results from each group and the corresponding control was determined by unpaired *t* test or ANOVA. All the results are expressed as means ± SEM from at least 3 independent experiments.

## Results and discussion

### Synthesis and characterization of CB[6] @AuNRs

HA was selected for the immobilization of the macrocycle CB[6] because of its: long biomedical history [39], non-immunogenicity and biodegradability [40], many hydroxyl groups for chemical modification, and recognition by the CD44 receptor expressed by some cells, in particular cancer cells [40]. The synthesis of the CB[6] HA conjugate was obtained by the photoreaction of thiol-functionalized lower molecular weight HA with the (allyoxy)_12_CB[6] as shown in Fig. 1b [41]. The degree of CB[6] substitution (DS) in the polymer was determined by ^1^H NMR (Additional file 1: Figure S1) and the results showed a DS of 8 ± 1 mol % of CB[6] in HA, as calculated by the integration of the polymer acetoamido signal and the CB[6] signals between 5.25 and 6.0 ppm.

**Fig. 2:**
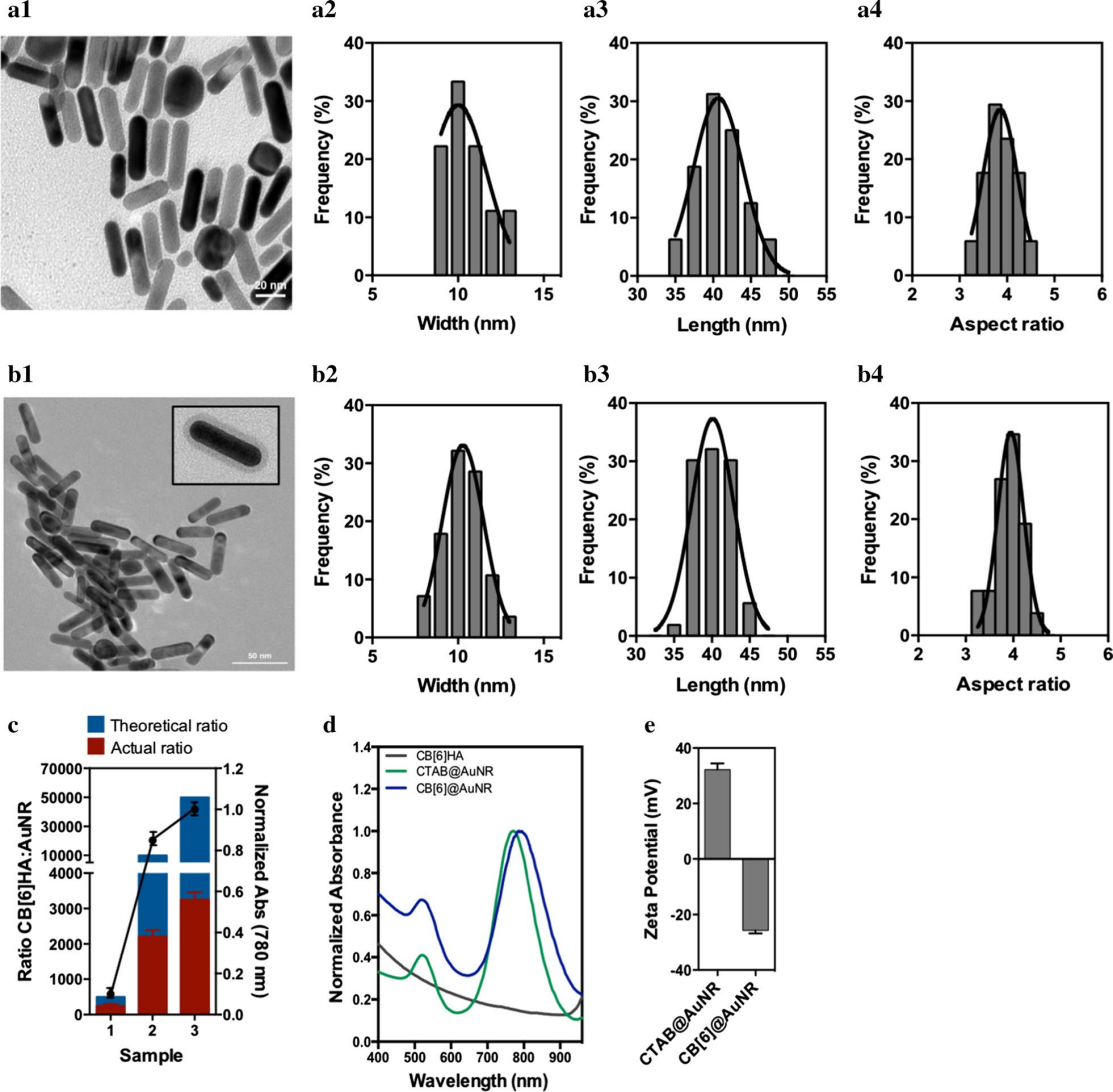
**a** Characterization of AuNRs before and after conjugation with CB[6] HA. **a, b** Representative TEM images of CTAB@AuNR (**a.1**) and CB[6] @AuNR (**b.1**). AuNR width (**a.2** and **b.2**), length (**a.3** and **b.3**) and aspect ratio (**a.4** and **b.4**). The AuNRs showed an average width of 10.3 ± 0.2 nm, an average length of 40.2 ± 0.4 nm and an aspect ratio of 3.9 ± 0.1. Results are average ± SEM (*n* = 50). **c** Amount of CB[6] HA at the surface of AuNR in different CB[6] HA:AuNR ratios determined by the anthrone assay (~ 3252 ± 354 polymer chain/NR for the theoretical ratio of 50.000:1). Absorbance at 780 nm was normalised by the absorbance of AuNRs before ligand exchange. Results are average ± SEM (*n* = 3). Absorbance spectra (**d**) and zeta potential (**e**) of AuNRs before (CTAB@AuNR) and after conjugation with CB[6] HA (CB[6] @AuNR)

The AuNRs were synthesized through a seed-mediated method with CTAB as a stabilizer. The AuNRs had an average length of 40.2 ± 0.4 nm and width of 10.3 ± 0.2 nm, an aspect ratio of 3.9 ± 0.1 and showed a plasmon resonance band at 780 nm (Fig. 2a, d). The removal of CTAB from the longitudinal facet of the rod is challenging and thus the coverage of AuNRs with CB[*n*] is not trivial [42, 43]. Previous studies have used a surfactant-free wet chemistry with the subsequent formation of gold nanostars instead of well-defined nanorods [13, 43]. However, the synthesis of gold nanostars with CB[*n*] leads to a decrease in the binding strength of the host–guest complex, since one of the macrocycle portals (able to stabilize guest molecules) is attached to the gold nanostar surface [13, 43]. Therefore, in the current study, we conjugated the macrocycle to a thiol-modified HA followed by the conjugation of the polymeric derivative to the surface of AuNR. One-pot ligand exchange was the most straightforward approach, and therefore CB[6] HA was added directly to an aqueous suspension of CTAB@AuNRs. To investigate the amount of CB[6] HA polymer required for conjugation, we conducted the reaction with different ratios of polymer to AuNRs, ranging from 500 to 50.000, while the concentration of AuNRs was maintained constant (Additional file 1: Figure S2A). Whereas ratios CB[6] HA:AuNRs of 500:1 and 10,000:1 resulted in incomplete exchange and concentration losses due to aggregation of the nanorods (Fig. 2c), stable AuNRs where obtained by using a ratio 50.000:1. To quantitatively evaluate the amount of CB[6] HA polymer chains in the surface of each AuNR, we used the anthrone assay (Fig. 2c). The results showed the presence of 3252 ± 354 polymer chains per AuNR (theoretical ratio 50.000:1), which is in line with results described in the literature for other ligands [44]. To confirm the CTAB replacement by CB[6] HA (from here on denoted as CB[6] @AuNR), the AuNR surface ligand composition was characterized by TEM, UV–Vis, zeta potential, FTIR and chemical analyses.

TEM images of CB[6] @AuNR showed the presence of AuNRs with an aspect ratio of around 3.9, and thus similar to the initial aspect ratio of AuNRs before conjugation with CB[6] HA polymer (Fig. 2b). CB[6] @AuNRs were also characterized by UV–Vis analyses (Fig. 2d) and the same two distinct absorbance peaks were observed at 530 nm and 780 nm after ligand exchange, which corresponded to the transverse and longitudinal surface plasmon resonances, respectively. Dynamic light scattering analysis of the AuNRs showed an increase of 17 ± 3 nm in length and 8 ± 2 nm in width (Additional file 1: Figure S2B, C) [45], while the zeta potential changed from +31 ± 3 mV with CTAB as a ligand to − 25 ± 2 mV after CB[6] HA ligand exchange (Fig. 2e). Although zeta potential is the most widely used characterization technique to confirm the surface modification of CTAB-capped AuNRs, it does not give information regarding CTAB removal. Therefore, FTIR spectroscopy was used to confirm the ligand exchange process. The FTIR spectra showed the disappearance of the absorptions at 2913 and 2845 cm^−1^ caused by the C-H stretching vibration of methyl and methylene groups of CTAB, confirming the removal of CTAB from AuNR surface (Additional file 1: Figure S2D). Conversely, the bands at 1740 and 1435 cm^−1^ attributed to C=O and C–N vibration, respectively, confirmed the immobilization of CB[6] HA in the surface of AuNR. Finally, the stability of CB[6] @AuNRs was studied in cell culture RPMI medium for 3 days (Additional file 1: Figure S2E). Our results showed that the colloidal suspension had an increase of 30 ± 2% in size with time, however, the number of nanoparticles in suspension remained the same.

Taken together, we have developed a new chemical strategy to coat AuNRs with macrocycles, specifically CB[*n*]. The strategy comprised two steps: (i) conjugation of thiolated HA with vinyl-modified CB[6] and (ii) conjugation of CB[6] HA to AuNRs by ligand exchange. Although AuNRs coated with HA have been described previously [46], the formulation described here is the first to have a macrocycle for host–guest chemistry.

### Light-triggerable release of small molecules from CB[6] @AuNRs

**Fig. 3:**
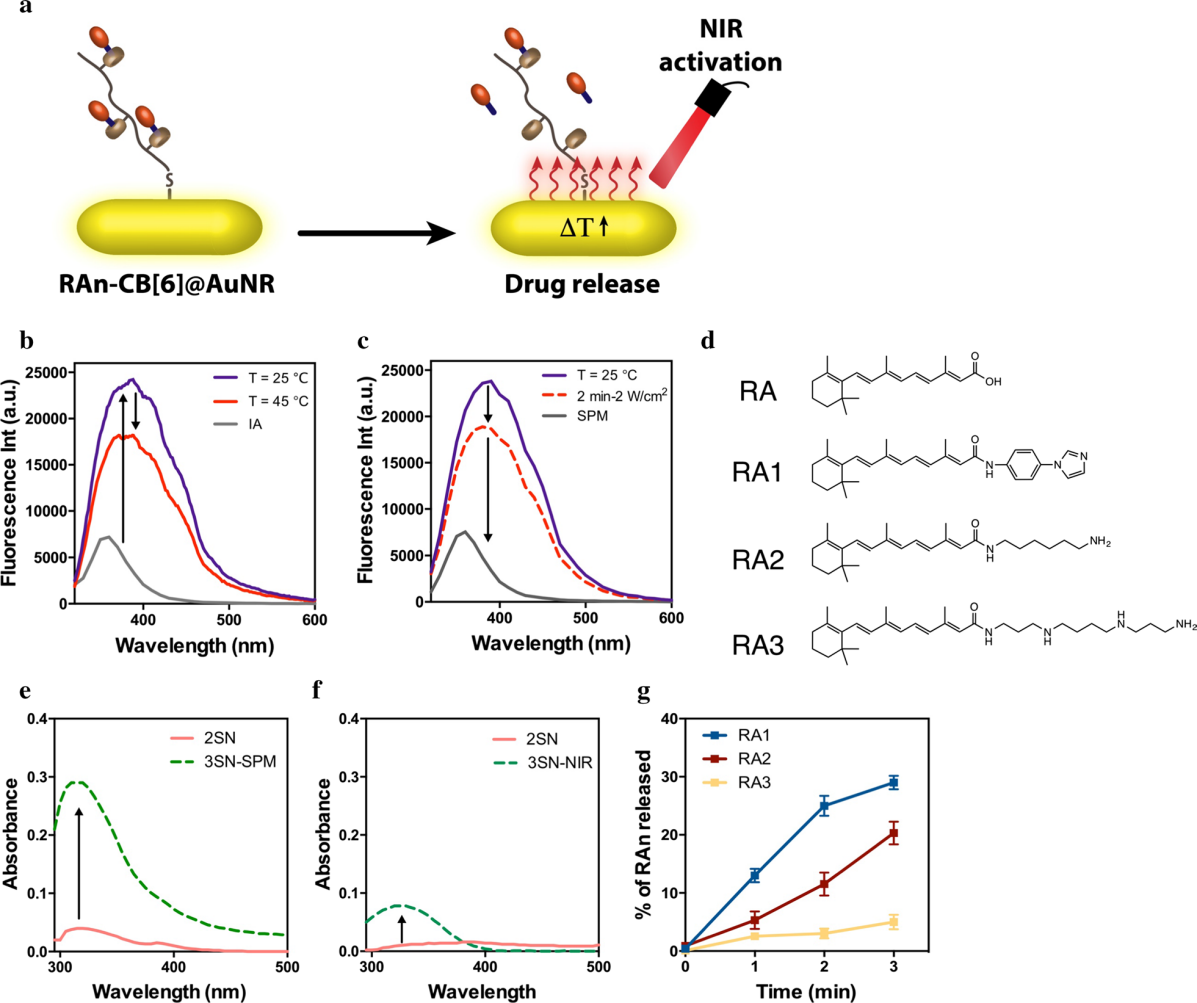
Release profile of IA and RA from CB[6] @AuNRs. **a** Schematic representation of the cargo release after irradiation the AuNRs with near-infrared light (780 nm, 2 W/cm^2^). **b** Representative fluorescence spectra of IA complexed with CB[6] @AuNR (50 µg/mL) at different temperatures. **c** Representative fluorescence spectra of IA complexed in CB[6] @AuNR (50 µg/mL) upon irradiation with NIR light for 2 min or after adding a competitive ligand (spermine (SPM), 0.5 mM)) to displace IA. **d** Chemical structures of the RA conjugates. **e** RA1-CB[6] @AuNRs (10 µg/mL) were irradiated by NIR laser, the suspension centrifuged two times (2SN), the supernatant collected and the absorbance measured. At the end, the formulation was suspended in water and a competitive ligand (SPM 0.5 mM) was added to displace RA1 from AuNRs (3SN-SPM). **f** Quantification of RA1 released after irradiation with NIR light. After irradiation, the suspension was immediately centrifuged, the supernatant was collected (3SN-NIR) and the amount of RA1 was determined using a calibration curve of RA1. **g** Percentage of RA1, RA2 and RA3 release upon irradiation the CB[6] @AuNRs (10 µg/mL) for different times. Results are expressed as average ± SEM (*n* = 3)

Next, we evaluated CB[6] @AuNRs as a drug release system (Fig. 3a). For proof of concept, we loaded the CB[6] @AuNRs with a fluorescent dye, N-(4-aminophenyl)imidazole (IA), with affinity to CB[6]. IA forms a stable 1:1 host–guest complex with CB[6] (*K*_*a*_ = 1.0 × 10^5^ M^−1^ at 25 °C), which upon complexation increases the fluorescence [47]. As expected, the fluorescence of IA increased when IA was added to an aqueous suspension of CB[6] @AuNRs, indicating the complexation of the guest by the macrocycle (Fig. 3b). When the formulation was heated at 45 °C the fluorescence of IA decreased due to the displacement of IA from CB[6] (Fig. 3b).

Similar results were obtained when IA-CB[6] @AuNRs suspended in water were exposed to a NIR laser (780 nm, 2 W/cm^2^, 2 min) (Fig. 3c). The exposure of AuNRs to NIR light causes collective oscillation of surface electrons, which in turn results in a localized heating phenomenon. In agreement with the temperature experiment, the results showed a decrease in the fluorescence intensity of IA, indicating the displacement of the dye from the cavity of CB[6] (Fig. 3c). In both experiments (temperature and NIR activation) the complete displacement was not achieved, at least in the conditions tested, since the addition of a competitive ligand, spermine (SPM; *K*_SPM-CB[6]_ ≈ 10^12^ M^−1^), displaced additional ligand from the AuNRs.

Next, we loaded the CB[6] @AuNRs with RA. Since RA was not complexed by the small cavity of CB[6], three RA conjugate with amines were synthesized (Fig. 3D). The high selectivity and affinity that this type of macrocycles has towards cations through the ion–dipole effect, along with its ability to complex alkyl chains within its hydrophobic cavity, is well known [16]. The three guests conjugated with the RA were selected in order to obtain different binding affinities with CB[6] (Additional file 1: Figure S3A–C). The activity of the RA derivatives was evaluated in a reporter cell line that expressed luciferase upon activation of RAR-α receptor by RA. The results showed a similar activity between RA1 and unmodified RA whereas RA2 showed a 50%-decrease in activity and RA3 a 70%-decrease as compared to the unmodified RA (Additional file 1: Figure S3D).

The binding affinity for the host–guest complex formation was estimated to be up to 10^5^ M^−1^ for RA1, 10^6^ M^−1^ for RA2 and 10^12^ M^−1^ for RA3 [48]. The amount of loaded RA conjugate was determined by calculating the difference between the amount of RA added to CB[6] @AuNRs and the amount of RA determined in the supernatant after centrifugation at 37 °C. In addition, the total amount of RA derivatives complexed in the CB[6] @AuNRs was also confirmed by adding an excess of a competitive ligand, SPM (0.5 mM) (Fig. 3e). UV–Vis was used to quantify the RA conjugate and the results showed that for the RA conjugate with the smaller binding affinity with CB[6], RA1, up to 50 µg of RA was loaded per mg of CB[6] @AuNR whereas for RA2 and RA3, the loading was 55 µg and 65 µg, respectively. The amount of RA conjugate released by CB[6] @AuNRs upon irradiation with NIR light was also evaluated. Without irradiation, the absorbance of RA1 in the supernatant was negligible, while after NIR irradiation, there was significant increase of the RA1 absorbance in the supernatant (Fig. 3f). Indeed, thirty percent of RA1 was released after 3 min exposure to NIR laser with power of 2 W/cm^2^ (Fig. 3g) which corresponded to an increase of 10 ± 1 °C in the CB[6] @AuNR suspension (Additional file 1: Figure S4A). Importantly, the irradiation of the RA1-CB[6] @AuNR suspension for 3 min did not affect the stability of AuNRs in suspension (Additional file 1: Figure S4B). The percentage of RA2 and RA3 released was 20% and 7%, respectively, due to higher binding affinity to CB[6] than RA1. We also investigated whether a NIR laser (1 W/cm^2^) could cross mouse tissues with different thickness (above the limit for UV radiation) and origins and trigger the release of RA1 from RA1-CB[6] @AuNR (Figure S4C). Our results show that up to 15% of RA1 was released being the extent dependent in the thickness and origin of the tissue. In a parallel set of experiments, we evaluated the binding affinity between RA1 and CB[6] at different temperatures (Additional file 1: Table S1). The release of 30% of RA1 from CB[6] @AuNRs required an increase of 15 °C in temperature. This temperature was higher than the one observed after laser irradiation (≈ 10 °C) for the same level of RA1 release (i.e. 30%), which suggested that the temperature reached at the surface of the AuNRs was higher than the bulk temperature, as observed in a study reported recently by us [11].

Overall, we have prepared a nanoformulation that releases RAn after activation by NIR while maintaining its stability in suspension. The displacement of the guest from the host is induced by the heating of the nanocarrier caused by its NIR activation. For the subsequent studies, we have used RA1 and RA2 since RA3 had relatively low activity.

### Cellular uptake and intracellular trafficking

Next, we evaluated the interaction of our formulation with human cells. Recently, we have shown that formulations able to release intracellularly small molecules may modulate the differentiation of leukemic cells and reduce the progression of blood cancers [26]. Therefore, we first evaluated the cytotoxic profile of RA1-CB[6] @AuNRs (we used RA1 for proof of concept) against human leukemia cells, i.e., human bone marrow APL NB4 cells, using an ATP assay. The results showed that RA1-CB[6] @AuNRs had no substantial effect on leukemia cells for concentrations up to 50 µg/mL (Additional file 1: Figure S5A). We also evaluated the cytotoxic effect of NIR light in the cells. The cells where incubated for 4 h with RA1-CB[6] @AuNRs (50 µg/mL), washed to remove the non-internalized AuNRs, and then irradiated during different times with NIR light at 2 W/cm^2^ (Additional file 1: Figure S5B). The results showed low cytotoxic effect, below 20% with up to 2 min of irradiation and are in line with other reported studies [11, 49].

**Fig. 4:**
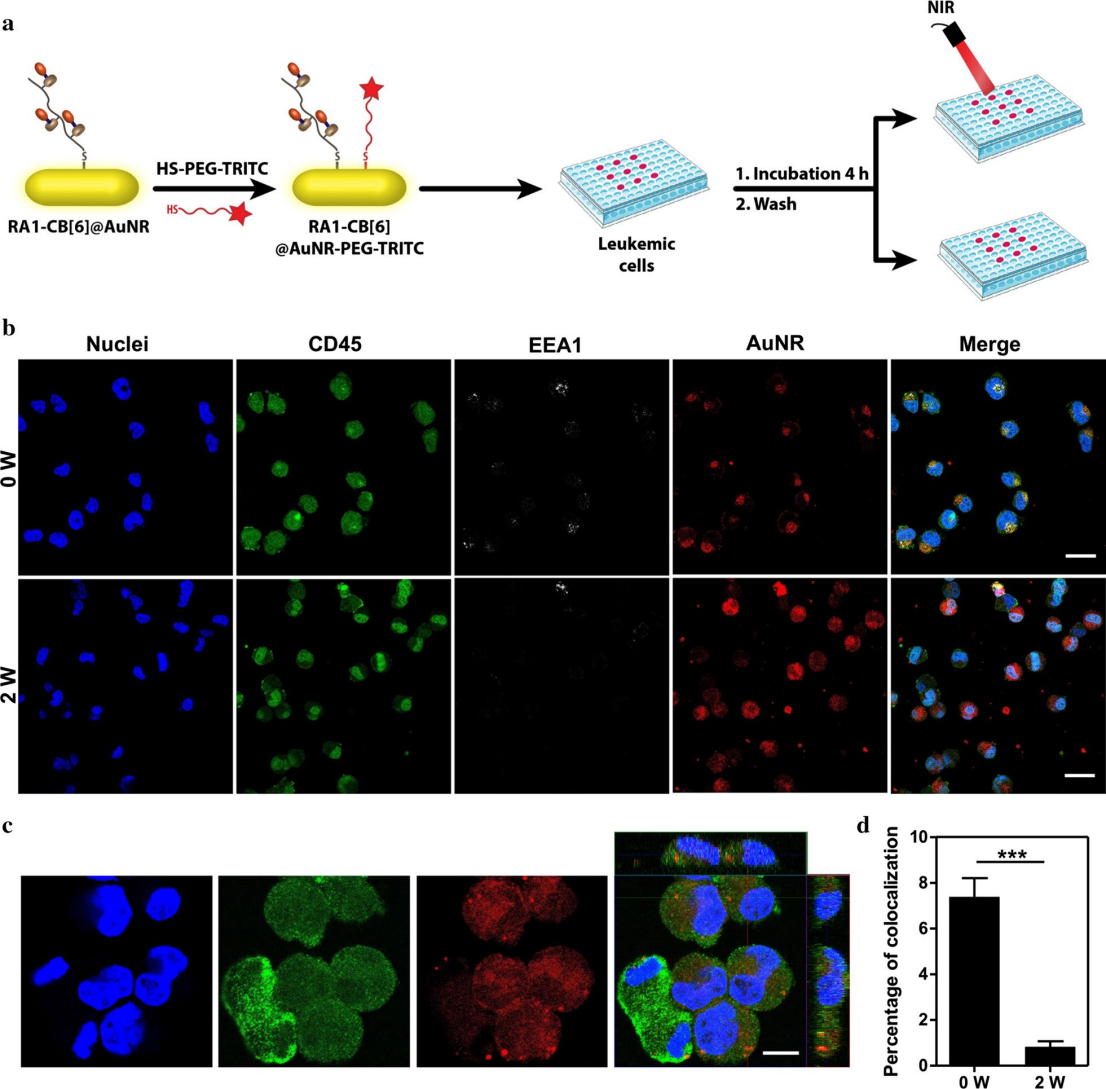
Intracellular trafficking of RA1-CB[6] @AuNRs as evaluated by immunocytochemistry. **a** Schematic representation of the experiment. **b** Confocal images of leukemic cells incubated with RA1-CB[6] @AuNR-PEG-TRITC. Cells were incubated for 4 h with AuNR (50 µg/mL), washed to remove the non-internalised AuNRs, added new cell culture media, irradiated for 2 min with a 780 nm laser (power: 2 W/cm^2^) and finally fixed with PFA (4%). The cell membrane was immunostained for CD45, while endosomes were immunostained for EEA1. White scale bar corresponds to 30 µm. **c** Intracellular localization of RA1-CB[6] @AuNRs before NIR light irradiation was confirmed by a Z-stack scan. Scale bar corresponds to 10 µm. **d** The co-localization between RA1-CB[6] @AuNR-PEG-TRITC and early endosomes before and after irradiation was quantified by image analyses. Results are average ± SEM (*n* = 3). Statistical analysis was performed using a t-test. ***Denotes statistical significance (P < 0.001)

To investigate the uptake of RA1-CB[6] @AuNRs in NB4 cells, the intracellular distribution of the formulation was examined. RA1-CB[6] @AuNRs were chemically conjugated with tetramethylrhodamine (TRITC) thiol-PEG and incubated with NB4 cells for 4 h. Then, the colocalization between RA1-CB[6] @AuNR-TRITC and early endosomes (immunostained for EEA1 epitope) was observed using confocal laser scanning microscopy (CLSM) (Fig. 4a). Most of the RA1-CB[6] @AuNR-TRITC escaped the early endosomes without light activation, and were located in the cell cytoplasm (Fig. 4b–d). The exposure of the cells to NIR light increased the amount of AuNRs in the cell cytoplasm. The amount of RA1-CB[6] @AuNR per cell was determined by ICP-MS for different incubation times (Additional file 1: Figure S5C). The results showed a rapid internalization of the RA1-CB[6] @AuNRs, since the internalization after 1 h of incubation was similar to the one obtained after 24 h.

Taken together, the formulation RA1-CB[6] @AuNRs had no significant cytotoxic effect on leukemic cells for concentrations up to 50 µg/mL and was rapidly (≈ 1 h) internalized by the cells and accumulated in cell cytoplasm. Our results are aligned with previous studies showing that AuNRs were not cytotoxic against leukemic monocyte macrophages for concentrations up to 40 µg/mL [50]. Interestingly, the uptake of AuNRs observed in NB4 cell line was higher (≈ 7 pg/cell) than the one previously observed in leukemic cells (≈ 0.8 pg/cell) [50] and this might be due to differences in AuNR surface composition and differences in cell types. In contrast to previous studies [51], our results indicate that RA1-CB[6] @AuNRs escape the endolysosomal compartment. NB4 cells (acute myeloid subtype 3, promyelocytic) express CD44, a transmembrane glycoprotein that is a receptor of HA [52]. It is possible that the interaction of HA with CD44 receptor potentiates AuNR cell uptake and endolysosomal escape, as it was observed recently with other AuNRs coated with HA [46]. This is an issue that deserves further investigation in the near future.

### Intracellular activation of RAn-CB[6] @AuNRs

**Fig. 5:**
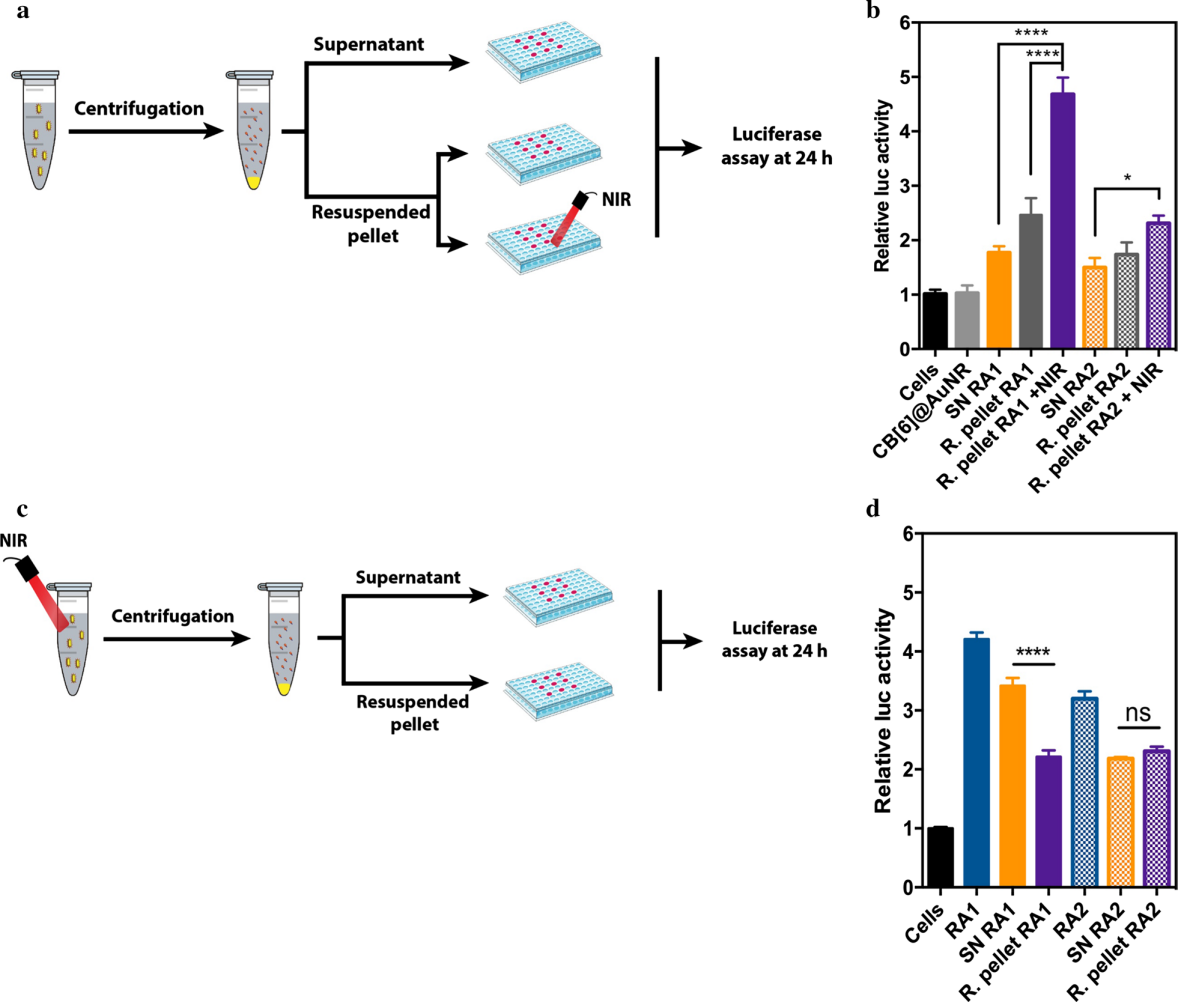
Biological activity of RA released from RAn-CB[6] @AuNRs outside leukemia cells. **a** Schematic representation of the in vitro release experiment without NIR light exposure outside cells in a leukaemia reporter cell line. **b** Activity of RA1 or RA2-CB[6] @AuNR (50 µg/mL) supernatant (SN) without irradiation to assess the leakage from CB[6] @AuNRs and activity of resuspended pellet (R. pellet) without or with NIR light activation after 4 h of incubation (780 nm, 2 min, 2 W/cm^2^). **c** Schematic representation of the in vitro release experiment with NIR light exposure outside cells in a leukaemia reporter cell line. RAn-CB[6] @AuNRs (50 µg/mL) were irradiated by a NIR laser (780 nm, 2 W/cm^2^) for 2 min after which the suspension was centrifuged and the activation of the leukemic reporter cell line (luciferase measurements) was measured both in the supernatant (SN) and resuspended AuNR pellet (R. pellet). **d** Activity of RA1 or RA2 in the supernatant and resuspended pellet evaluated by the leukemic reporter cell line. Cells were cultured with soluble RA1 (1 µM), RA2 (1 µM), SN and resuspended pellet for 24 h, followed by luciferase luminescence reading. In B and D, results are average ± SEM (*n* = 3). Statistical analysis was performed using One-Way Anova followed by Tukey’s post hoc test. *, **** denotes statistical significance (P < 0.05, P < 0.0001)

To investigate the biological impact of RAn-CB[6] @AuNRs in leukemic reporter cells, we initially performed the release of RA conjugates outside the cells and then incubated the cells with the released contents (Fig. 5). With this in mind, RAn-CB[6] @AuNRs were either exposed or not to a NIR laser (2 W/cm^2^) for 2 min after which the suspension was centrifuged and the following experimental groups were evaluated in the human bone marrow APL NB4 reporter cell line: (i) supernatant and resuspended pellet collected from RAn-CB[6] @AuNRs without NIR activation (Fig. 5a); (ii) supernatant and the resuspended pellet collected from RAn-CB[6] @AuNRs with NIR activation (Fig. 5c). In all cases, cells were incubated for 4 h with each solution/formulation, washed, cultured for additional 20 h and then the levels of luciferase quantified.

Our results showed that CB[6] @AuNRs released a relatively small amount of RAn in the absence of NIR activation confirming the stability of the host–guest interaction (Fig. 5b). Moreover, the resuspended pellet showed low relative luciferase activity without NIR activation, while irradiation with NIR light the cells incubated for 4 h with the resuspended pellet lead to a high luciferase activity (Fig. 5b). On the other hand, CB[6] @AuNRs released a significant amount of RA1 or RA2 after NIR activation (Fig. 5d). Due to different binding affinities between RA1 or RA2 to CB[6] @AuNR, the supernatant RA1 showed higher luciferase activity relatively to the resuspended pellet, as compared to the supernatant with RA2.

**Fig. 6:**
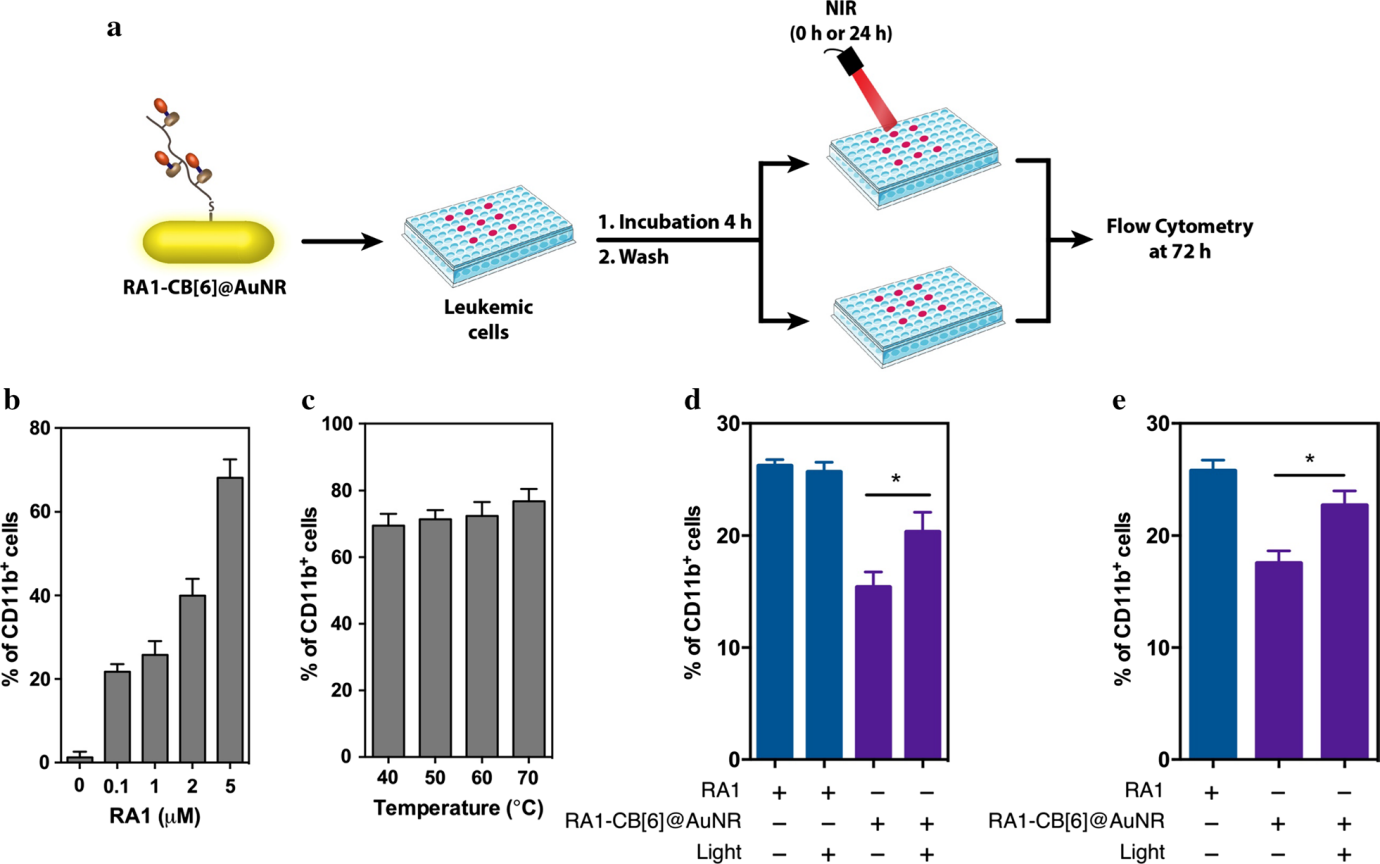
Biological activity of RA released from RAn-CB[6] @AuNRs within leukemia cells. **a** Schematic representation of the assay. Leukemia cells were treated with AuNR (50 µg/mL) for 4 h, washed with PBS to remove the AuNRs that were not internalised, resuspended in cell culture media, exposed to NIR light for 2 min (780 nm, laser power 2 W/cm^2)^ and cultured for 68 h. At 72 h the cells were analysed by flow cytometry. **b** Myelocytic differentiation of NB4 cells cultures by varying the amount of RA1 and analysed at 72 h. **c** RA1 (5 µM) was heated at different temperatures during 5 min before adding to NB4 cells. Cell differentiation at 72 h as assessed by the expression of CD11b^+^ by flow cytometry. **d**, **e** Percentages of CD11b^+^ in leukemia cells differentiated for 72 h when cultured with soluble RA1 (1 µM) for the entire duration of the experiment, or incubated for 4 h with RA1-CB[6] @AuNR, washed and immediately irradiated at 0 h (**d**) or at 24 h (**e**). Results are expressed as Mean ± SEM (*n* = 3). Statistical analysis was performed using One-Way Anova followed by Tukey’s post hoc test. * denotes statistical significance (P < 0.05)

Next, we monitored the biological impact of the intracellular release of RA1 from RA1-CB[6] @AuNRs in a leukemic cell line (Fig. 6a). RA1-CB[6] @AuNRs was selected for these tests in detriment of RA2-CB[6] @AuNRs due to its high activity, based on the results obtained on the reporter cell line. The intracellular release of RA1 was followed by the expression of myeloid maturation marker CD11b^+^, a protein involved in the regulation of leukocyte adhesion and migration, using flow cytometry. Initially, we evaluated the impact of different concentrations of soluble RA1 in the differentiation of leukemic cells (Fig. 6b). Then, due to localized heating on AuNRs surface upon NIR irradiation, we studied the influence of irradiation time on RA1. The results showed that heating RA1 (5 µM) for 5 min at different temperatures did not affect the percentage of cells expressing CD11b^+^ (Fig. 6c).

To assess the release of RA1 from the CB[6] @AuNRs, leukemic cells were transfected for 4 h, washed, activated or not by a NIR laser (2 min, 2 W/cm^2^), cultured for additional 68 h and finally characterized by flow cytometry. Although non-irradiated cells also induced RA signaling, the results showed higher percentage of cells expressing CD11b^+^ upon NIR light activation (Fig. 6d). Moreover, when the cells were irradiated 24 h after the washing step (Fig. 6e), similar results were obtained, indicating the temporal control release of RA within cells.

Overall, our results demonstrated the controlled release of RA derivatives within leukemic cells after NIR activation. Although not investigated, the formulation proposed here may be suitable for the intracellular delivery of more than one ligand, being the controlled release governed by the binding affinity of each ligand to the host and the temperature profile controlled by the irradiation exposure (i.e. ligands with low affinity will require low temperature for release, while ligands with high affinity will require high temperatures). During the preparation of the current work, a study has explored host–guest chemistry to release small molecules from nanocarriers upon NIR light activation within cells [13], however, the spatio-temporal controlled release of the drug from the macrocycle upon NIR activation was not demonstrated. In the previous study, the CB [7] was adsorbed to the surface of gold nanostars and thus not chemically conjugated to the surface of the nanocarrier like in the current study. Although the authors were able to demonstrate the encapsulation of an anticancer drug, the system was very leaky since 30% of the drug was released without light activation. It is anticipated that the direct attachment of the macrocycle to the gold nanostars decreased the binding strength of the guest to the host. Moreover, the authors did not show whether the release from the gold nanoshell was the drug or the drug interacting with the macrocycle. Although the intracellular release of small molecules has been reported by other light-disassembled nanoformulations [12], light-induced thermal de-hybridization of oligonucleotides from nanocarriers [9], light-induced thermal dissociation of small molecules from a polycationic linker attached to a AuNR [53], or light induced cleavage of a linker attached to the nanocarrier and having in the other extremity a small molecule [54], the strategy adopted in this study is relatively simple for the design of light-activatable systems given that it does not require significant alterations in the chemistry of the molecule of interest. Overall, this platform allows the immobilization of multiple molecules, with different chemical structures, to one single nanocarrier and, importantly for clinical applications, the activation by NIR light will facilitate tissue penetration.

## Conclusions

Here we report a formulation that integrates a NIR laser sensitive core and a polymeric coating composed by a macrocycle CB[6] for host–guest chemistry. This formulation may complex any small molecule conjugated with a guest ligand having affinity for the macrocycle. We demonstrated this concept with RA, which is a small molecule commonly used for therapeutic and regenerative medicine approaches. We showed that RA conjugated with different CB[6] ligands had different affinity to the formulation and, depending on the ligand chemistry, up to 65 µg of RA was immobilized per mg of AuNRs. Importantly, upon exposure to NIR laser the formulation was able to release approximately 30% of RA within 2 min. We demonstrated the biological effect of RA derivatives released intracellularly in a reporter cell line and on the expression of CD11b protein at the cell surface. We envision that this formulation might open new opportunities to tackle leukemic niches as recently demonstrated by us using a UV-light activatable formulation, which has a much lower tissue penetration [26].

## Supplementary information

**Additional file 1.** Data relative to the characterization of RA and AuNRs conjugates, cytotoxicity, AuNRs heating profile, internalization and binding affinities of RAn to AuNRs.

## Data Availability

All data generated or analyzed during this study are included in this published article and its additional information files.
