# Supplementary material for: A near infrared light-triggerable modular formulation for the delivery of small biomolecules
Source: J Nanobiotechnology. 2019 Sep 16;17:97. doi: 10.1186/s12951-019-0530-y (PMC6747754; doi:10.1186/s12951-019-0530-y)

**Additional Information**

**A near infrared light-triggerable modular formulation for the delivery of small biomolecules**

Vitor Francisco,^1^ Miguel Lino^1^ and Lino Ferreira^1,2*^

**^1^**CNC-Center for Neurosciences and Cell Biology, University of Coimbra, Coimbra 3004-517, Portugal

^2^Faculty of Medicine, University of Coimbra, 3000, Coimbra, Portugal

*Corresponding author: [lino@uc-biotech.pt](mailto:%20lino@uc-biotech.pt)


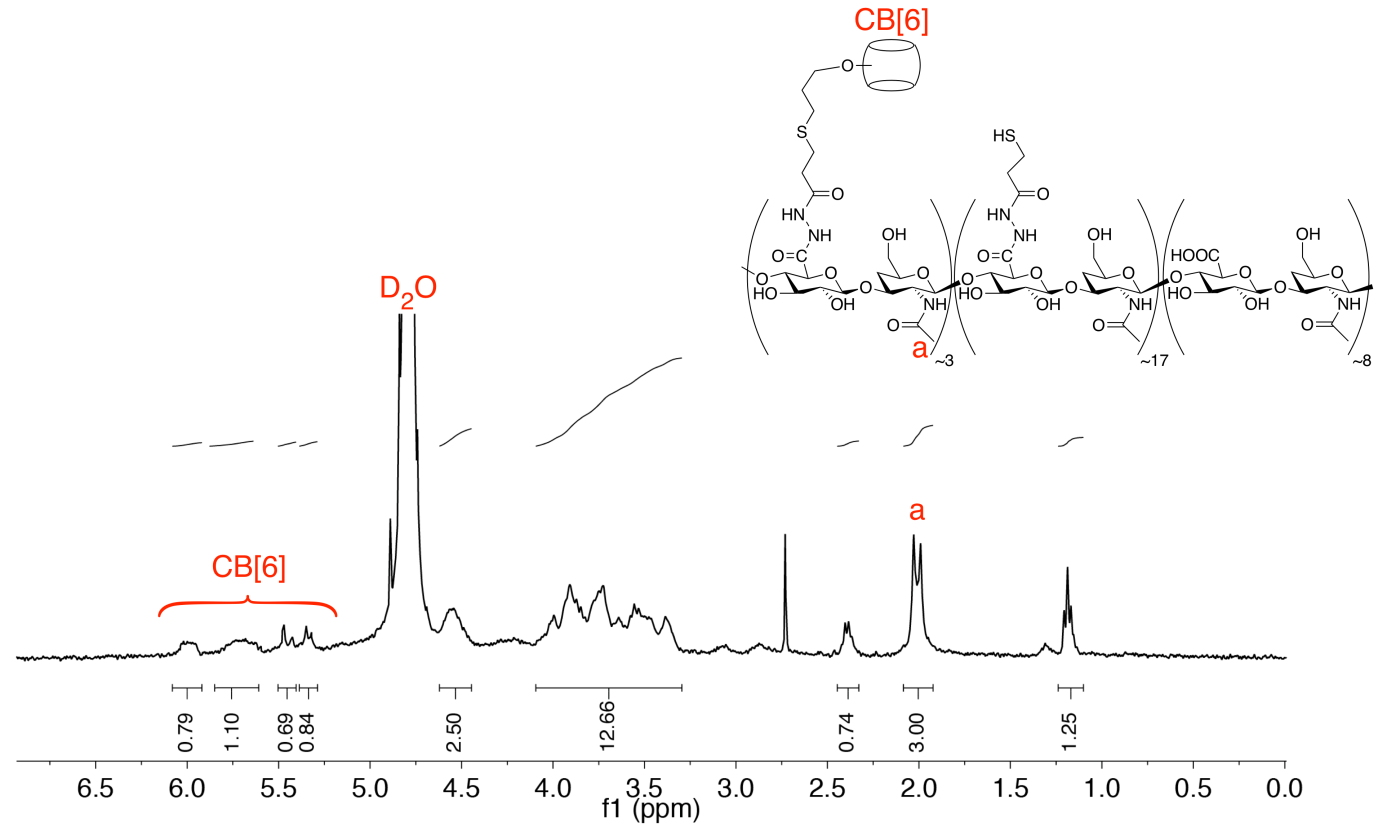


**Figure S1.** ^1^H-NMR spectrum in D_2_O of CB[6]HA confirming the modification of ca. 8 ± 1 mol% of HA units with (allyloxy)_12_CB[6].


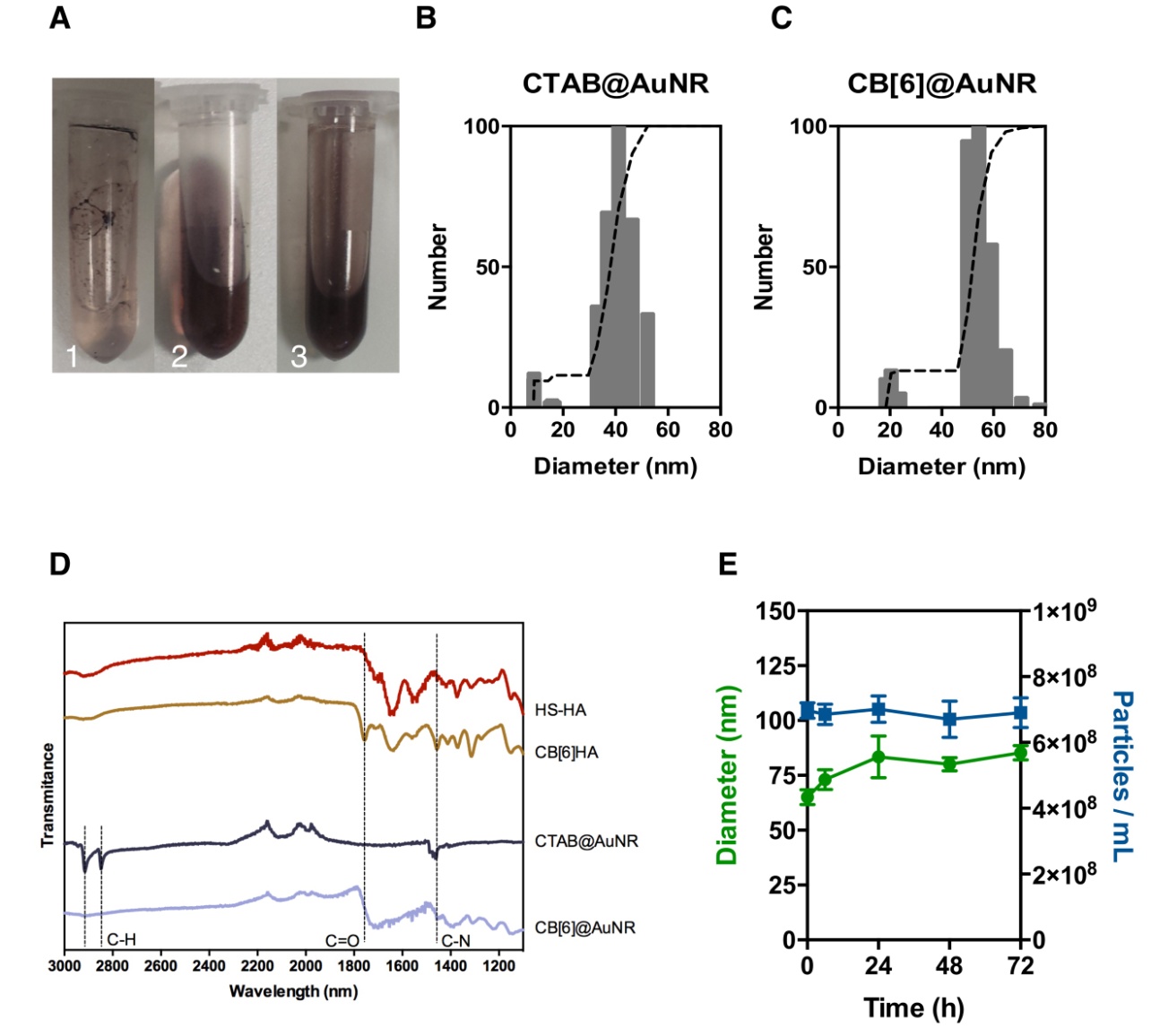


**Figure S2.** Characterization of the AuNRs before and after ligand exchange with CB[6]HA. (A) Different ratios of CB[6]HA to CTAB@AuNR in the ligand exchange experiment, 500:1 (1), 10000:1 (2) and 50000:1 (3). Dynamic light scattering of CTAB@AuNR (B) and AuNR after ligand exchange with CB[6]HA (C). (D) FT-IR spectra of thiol-modified HA (HS-HA), macrocycle modified HA (CB[6]HA), AuNR with CTAB ligand (CTAB@AuNR) and macrocycle modified AuNR surface (CB[6]@AuNR). (E) Stability of AuNR suspended in cell culture media (RPMI without FBS) overtime determined by nanoparticle tracking analysis. Results are expressed as Mean ± SEM (*n* = 3).


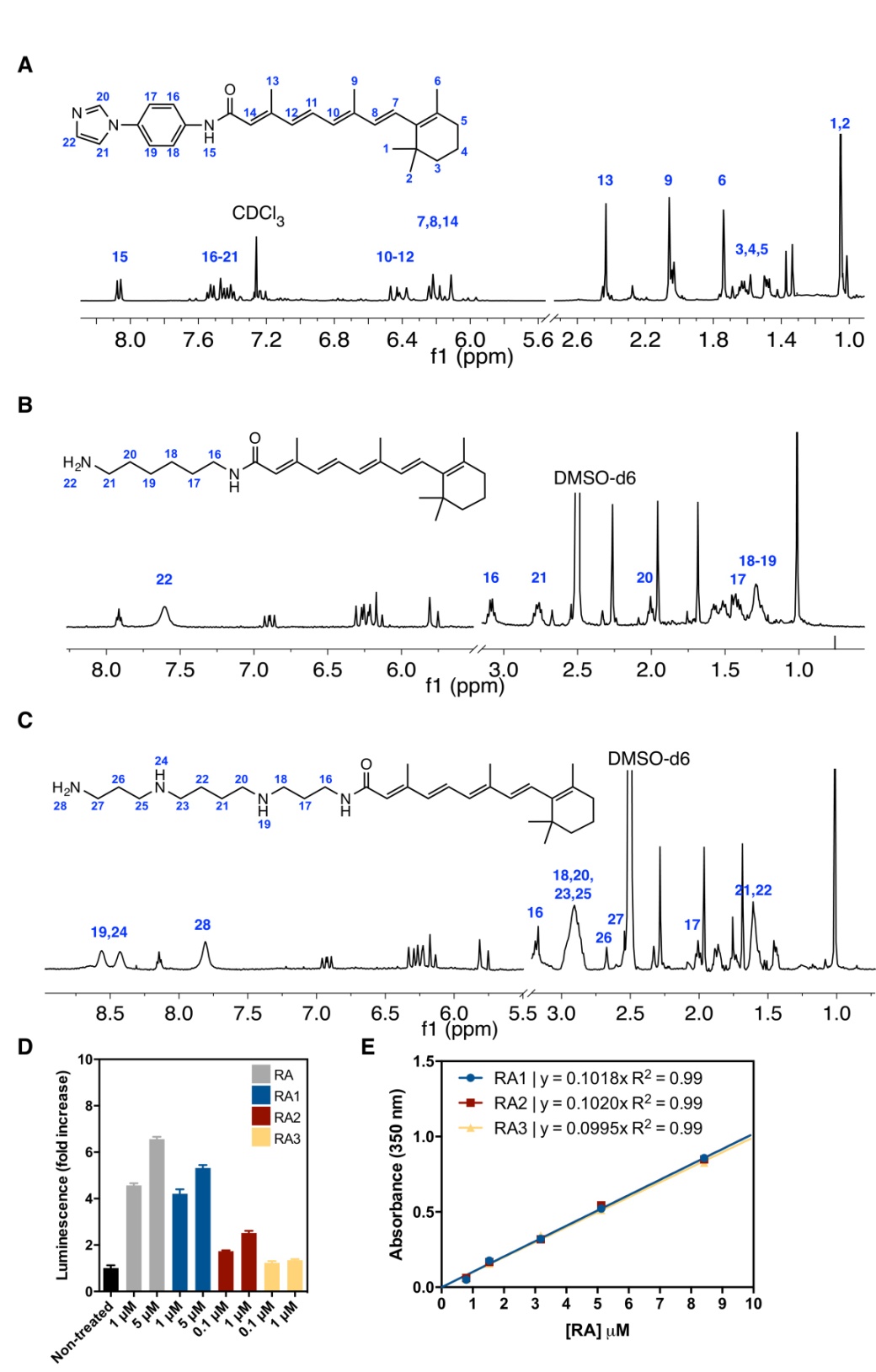


**Figure S3.** ^1^H NMR spectra of RA1 in CDCl_3_ (A), RA2 (B) and RA3 (C) in DMSO-*d*_6_ at 25 ºC. (D) Leukemic luciferase reporter cells were cultured with the four compounds for variable concentration followed by luciferase measurements at 24 h. The activation of RA-dependent signaling pathway was measured by luminescence. Results are expressed as Mean ± SEM (*n* = 3). (E) Linear calibration curve for the RAn conjugates.


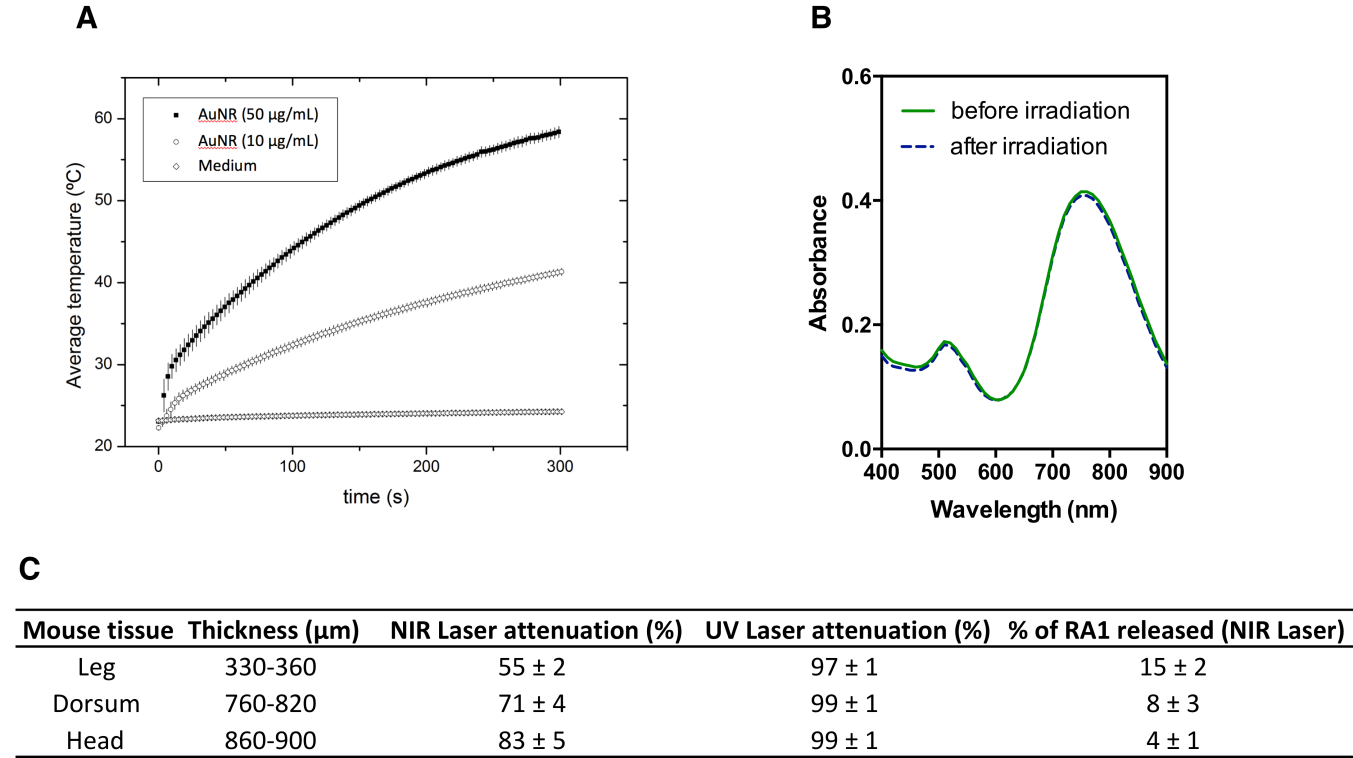


**Figure S4.** (A) Heating profile of RA1-CB[6]@AuNR suspensions (50 µg/mL) and (10 µg/mL) in RPMI-1610 medium, irradiated with a 780 nm laser (2 W/cm^2^) and measured using a FLIR SC650 infrared camera. Error bar shows the difference between the average and the min/max temperature values obtained at the well plate. (B) Absorbance spectra of RA1-CB[6]@AuNR (50 µg/mL) before and after laser irradiation (2 W/cm^2^) for 3 min. (C) NIR and UV-light laser attenuation with different mouse tissue. A 1.5 cm x 1.5 cm skin (thickness measured by a caliper) was placed between a microscope slide and a cover slip on a top of a thermal power sensor (Thorlabs s310c). The different tissues were then irradiated with NIR laser (780 nm, 1 W/cm2) or UV light (365 nm, 40 mW/cm^2^) during 1 min. Laser attenuation values were calculated by normalising against laser power values obtained with the empty microscope slide and the cove slip. Percentage of RA1 release upon irradiation CB[6]@AuNRs (10 µg/mL) with NIR laser (4 min, 1 W/cm^2^) placed below skin. Results are Mean ± SEM (*n* = 3).


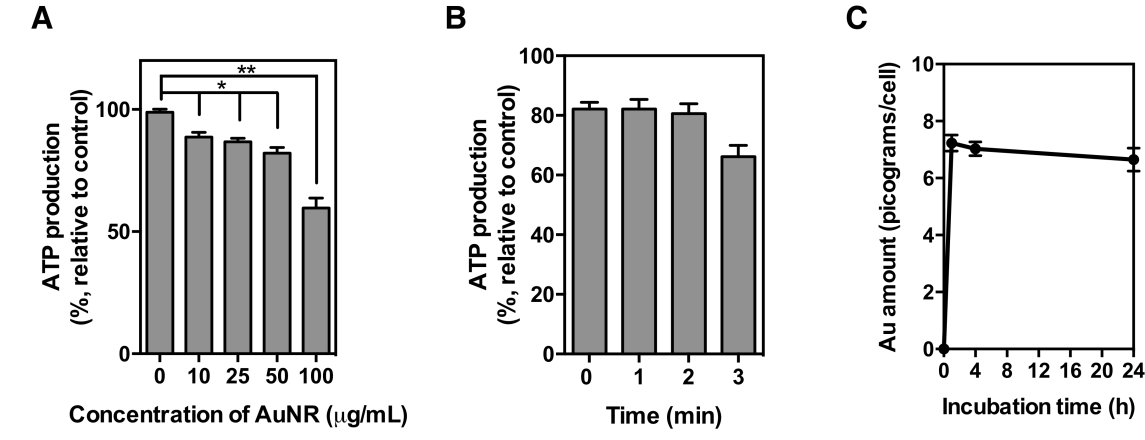


**Figure S5.** Cytotoxicity and uptake of RA1-CB[6]@AuNRs. (A) Leukemic cells were incubated with different concentration of RA1-CB[6]@AuNRs for 4 h, washed, incubated in cell culture media for 20 h after which cell viability was evaluated by an ATP kit. Results are expressed as Mean ± SEM (n = 3). *, ** denotes statistical significance (p<0.05; p<0.01) by one-way Anova followed by Tukey's *post-hoc* test. (B) Leukemic cells were incubated with RA1-CB6@AuNR (50 µg/mL) for 4 h, washed, irradiated with NIR light at 780 nm (2 W/cm2) for different times and cultured for additional 20 h before cell cytotoxicity evaluation by an ATP kit. Control was cells without exposure to RA1-CB6@AuNRs. Results are expressed as Mean ± SEM (n = 3). (C) Amount of Au in leukemic cells incubated with RA1-CB[6]@AUNR (50 µg/mL) for different times. After each incubation, cells were washed to remove the non-internalised AuNRs, centrifuged, resuspended and freeze-dried for ICP-MS analyses. Results are expressed as Mean ± SEM (*n* = 3).

**Table S1.** Binding constants *K*_a_ of RA1 and CB[6] at different temperatures at neutral pH.


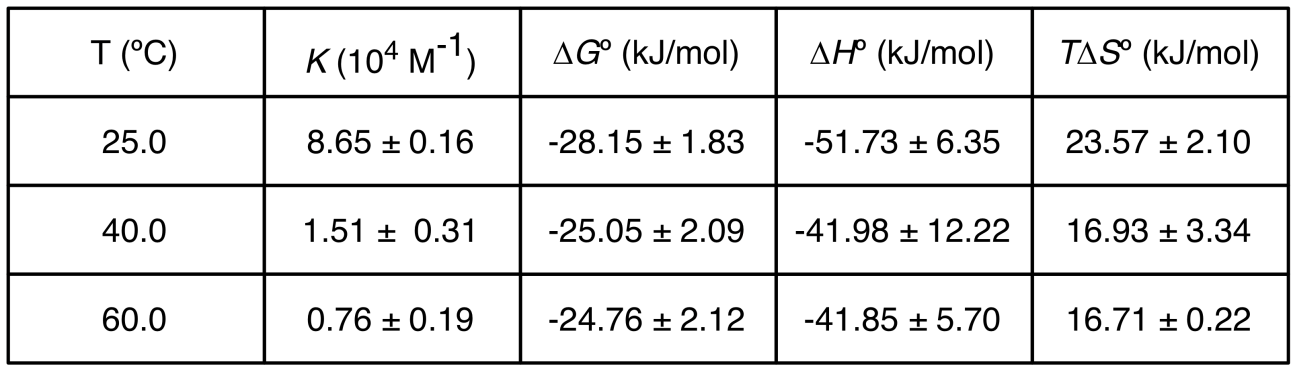

Supplement: Supplementary file 1 — Additional file 1. Data relative to the characterization of RA and AuNRs conjugates, cytotoxicity, AuNRs heating profile, internalization and binding affinities of RAn to AuNRs. [file 12951_2019_530_MOESM1_ESM.docx]
